# CXCL12 levels correlate with reduced stroke severity and lower risk of hemorrhagic transformation in stroke patients

**DOI:** 10.3389/fcvm.2026.1753482

**Published:** 2026-05-12

**Authors:** Fernando Ostos, Ana Moraga, María Isabel Cuartero, Sandra Vázquez-Reyes, Carolina Peña-Martínez, David Seoane, María Gutiérrez-Sánchez, Antonio Martínez-Salio, Blanca Diaz-Benito, Patricia Calleja, Alicia García-Culebras, María Ángeles Moro, Ignacio Lizasoain

**Affiliations:** 1 Department of Neurology and Stroke Center, Hospital 12 Octubre (imas12), Madrid, Spain; 2 Cellular Biology and Histology Department, Complutense Medical School, Instituto Investigación Hospital 12 Octubre (imas12), Madrid, Spain; 3 Neurovascular Research Unit. Pharmacology Department, Complutense Medical School, Instituto Investigación Hospital 12 Octubre (imas12), Madrid, Spain; 4 Neurovascular Pathophysiology, Centro Nacional Investigaciones Cardiovasculares (CNIC), Madrid, Spain; 5 Department of Psychology, Faculty of Health Sciences, Universidad Rey Juan Carlos, Madrid, Spain

**Keywords:** CXCL12, hemorrhagic transformation, ischemic stroke, neutrophil, stroke severity

## Abstract

**Background and purpose:**

Stroke is a leading cause of mortality and acquired disability. Acute-phase interventions like intravenous thrombolysis and mechanical thrombectomy have improved stroke prognosis, yet limitations persist, warranting novel therapeutic targets in cerebroprotection. Neutrophils play a key role in the post-stroke immune response, where recent studies have shown both detrimental and cerebroprotective roles. CXCL12 has emerged as a cytokine influencing neutrophil phenotype and potentially stroke severity, yet its effect in the acute phase remains controversial.

**Methods:**

In this prospective cohort study, 134 patients with ischemic stroke within 6 h of onset or with a wake-up stroke were included. We recorded clinical data, plasma CXCL12 levels, and neutrophil phenotype at admission, 24 h and 3 months post-stroke. We assessed stroke severity using NIHSS scores, ASPECTS scores, infarct volume at 24–72 h and functional outcomes at 3 months. Correlations between CXCL12, clinical severity, radiological findings, and functional outcomes were analyzed with regression models. A predictive model was developed using backward stepwise regression.

**Results:**

Higher admission CXCL12 concentrations were associated with lower radiological severity (ASPECTS) and reduced infarct volume at 24 h. Lower CXCL12 at 24 h was linked to increased hemorrhagic transformation risk, while no significant link was found with 3-month functional prognosis. Additionally, a positive correlation was observed between CXCL12 levels and plasma NETs markers. No associations were found between CXCL12 levels and neutrophil aging phenotype by nuclear segmentation.

**Conclusions:**

Our findings suggest CXCL12 levels are associated with acute stroke severity and with a lower risk of hemorrhagic transformation, though not with long-term functional outcomes. Further research is needed to elucidate the role of CXCL12 in stroke pathophysiology and to determine its potential relevance as a biomarker or therapeutic target.

## Graphical Abstract

**Figure:**
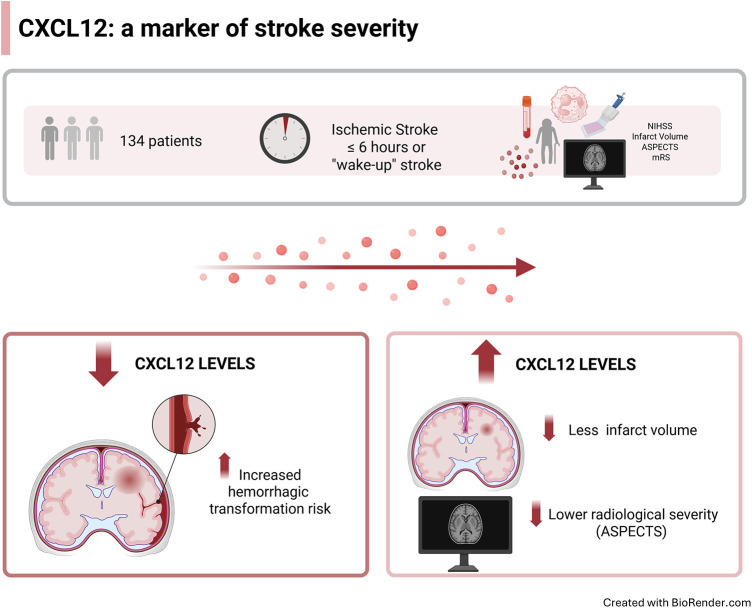

## Introduction

Stroke is one of the leading causes of mortality, acquired disability in adults and dementia worldwide (1). The prognosis of patients with ischemic stroke has improved significantly in recent decades, especially after the introduction of acute-phase treatments such as intravenous thrombolysis (2, 3) or mechanical thrombectomy (4). However, these treatments have important limitations that restrict their use to selected cases, and some patients do not show clinical improvement despite receiving them. Therefore, it is essential to identify new therapeutic targets in the field of cerebroprotection to improve the prognosis of stroke patients.

The immune response associated with ischemic stroke is a critical factor in the pathophysiology and resolution of stroke (5). Among the different cell types involved, the neutrophil is a key player in the inflammatory response in the first days after stroke. Although initially only deleterious effects were attributed to neutrophils, it has been shown that there are also neutrophils with cerebroprotective functions associated with the resolution of brain inflammation (6–9).

Chemokine CXC ligand 12 (CXCL12), previously known as stromal cell-derived factor-1 (SDF-1), is a chemokine involved in leukocyte homing and trafficking, and angiogenesis (10). It primarily mediates its functions through two receptors: CXCR4 and CXCR7. Its influence on the migration of neutrophils into the bloodstream and its role as a chemoattractant for immune system cells may modify the prognosis of patients with ischemic stroke.

In recent years, the role of CXCL12 on the phenotypic change of circulating neutrophils has also been described in a process known as neutrophil aging (11). Neutrophil aging has a circadian pattern and may influence the response to inflammatory, infectious, or vascular injury. Neutrophils entering circulation, known as "fresh" neutrophils, exhibit distinct membrane marker expression and nuclear morphology (12). In contrast, aged neutrophils are characterized by a hypersegmented nucleus (11). Furthermore, neutrophil phenotype can significantly influence neutrophil activity, including the formation of neutrophil extracellular traps (NETs) (13).

Previous studies on the influence of CXCL12 and NET formation after stroke have been insufficient and yielded contradictory results. The connection between CXCL12, phenotypic changes in circulating neutrophils, and their association with ischemic stroke severity remains unexplored. Understanding this relationship could identify a potential therapeutic target for cerebrovascular pathologies.

## Methods

This was a prospective single-center cohort study, including patients diagnosed with ischemic stroke within 6 h of onset or “wake-up” stroke between March 2019 and July 2022. Patients with lacunar stroke, TIA or ischemic stroke without a visible ischemic lesion on CT scan or MRI or patients with previous pathologies or treatments that could modify the immune response (such as recent infection, oncologic or active rheumatologic diseases) were excluded.

We prospectively recorded age, sex, history of hypertension, diabetes mellitus, dyslipidemia, atrial fibrillation, previous ischemic stroke or transient ischemic attack, previous hemorrhagic stroke, smoking status, stroke onset (known, wake-up stroke, unknown), previous functional status using the modified Rankin Scale (mRS), stroke subtype (TOAST criteria) (14). Daytime was defined as the time 8:00 am and 8:00 pm, whereas nighttime was defined as 8:00 pm to 8:00 am.

Stroke severity was assessed using the National Institute of Health Stroke Scale (NIHSS) at admission (<6 h from stroke onset) and at 24 h, and functional outcome was evaluated at 3 ± 1 months using mRS. Good functional outcome was defined as a mRS score ≤ 2.

### Laboratory tests

Blood samples were collected at admission (<6 h), after 24 h (+/− 12 h), and if possible, after three months (+/− 1 month). Blood samples were obtained at 08:00h +/− 3 h to reduce circadian oscillations, except on admission, that was obtained at their arrival and before the acute treatment was performed.

Biochemistry (glucose, creatinine, C reactive protein) hematology (total erythrocytes, leucocytes, neutrophils, lymphocytes, monocytes and platelets), and coagulation test were assessed in the central laboratory of the hospital.

In each case, a 5 mL sample of venous blood was collected and preserved with EDTA (ethylenediaminetetraacetic acid) to obtain platelet-poor plasma and a blood smear.

#### Platelet-poor plasma

After separation of the 500 μL for the blood smear, two centrifugations were performed. The first centrifugation was done at 1000g for 10 min at 4 °C to obtain platelet-rich plasma. The second centrifugation was done at 2,000 g for 15 min at 4 °C to obtain platelet-poor plasma. The samples were stored at −80 °C until further analysis.

CXCL12 amounts were measured in plasma samples using a commercially available enzyme-linked immunosorbent assay (ELISA) kit according to the manufacturer's instructions (R&D Systems, Minneapolis, MN). Samples were analyzed in duplicate, and optical density was measured using a microplate reader. Concentrations were calculated from a standard curve generated with recombinant CXCL12 provided in the kit.

To ensure intra-assay consistency, a pooled plasma sample with CXCL12 concentrations within the linear range of the standard curve was included as an internal control on each plate. Assays were accepted only when the coefficient of variation (CV) between duplicates was below 10%, in accordance with the manufacturer's recommendations. When the coefficient of variation between duplicates exceeded 10%, the ELISA was repeated to ensure accuracy; samples were only excluded if variability remained above this threshold after repetition.

NETs markers (cell free DNA, myeloperoxidase (MPO), neutrophil elastase) were measured in plasma samples at admission, following the manufacturer's protocol (cfDNA: P11496 de Invitrogen, Oregón, USA; MPO DMYE00B de R&D Systems, Minneapolis, USA; Neutrophil elastase). The plasma concentration of NE and MPO does not follow a normal distribution. Therefore, logarithmic values were used in the analysis.

#### Blood smears

The 500 μL of whole blood obtained is transferred to a 2 mL Eppendorf tube. Then, 1 mL of lysis buffer (50 mmol/L Tris-HCl, pH 7.4, 150 mmol/L NaCl, 5 mmol/L CaCl_2_, 0.02% NaN_3_, 1% Triton X-100) is added to lyse the red blood cells in the sample, and it is kept at room temperature for 10 min. Subsequently, the sample is centrifuged at 1,800 rpm for 10 min at room temperature.

The supernatant is removed and the pellet is resuspended in 80 μL of phosphate-buffered saline (PBS 1X), with the following composition per mmol/L: NaCl 137, KCl 2.7, Na_2_HPO_4_ 10, KH_2_PO_4_, pH 7.4. The suspension is then distributed onto two slides. After air-drying, the slides are fixed with 4% paraformaldehyde (PFA) for 10 min and stored at −20 °C. Subsequently, the samples are stained with Giemsa and digitized using the AxioScan Z1 digitalizer (Zeiss®) at 40× magnification.

Blood smears were analyzed with Zeiss Zen Blue software (v. 3.7 L). For each smear, we identified 100 neutrophils and recorded the number of lobes in each nucleus. Neutrophils were categorized into three groups based on the number of nucleus segmentations: 3 or fewer, 4, and 5 or more (15, 16).

Neuroimaging was performed on arrival to determine ASPECTS score and collateral status, and at 24–72 h to determine infarct volume and major complications (including hemorrhagic transformation).

### Statistical analysis

Statistical analysis was conducted using STATA v.15.1 (Stata Corp LP, TX, USA) and PRISM v.8.0 (GraphPad Software Inc., USA) were used to design the graphs showing the results. Values of *P* < 0.05 were considered statistically significant. Missing data were handled using a complete-case analysis, observations with missing values were excluded from the regression models.

For descriptive analysis, we reported percentages for binary variables and mean and standard deviation for continuous variables and median and interquartile range for discrete variables. A univariate analysis to determine the correlation between CXCL12 and various clinical and radiologic severity variables was performed using linear, logistic or quantile regression as appropriate. Finally, a predictive study was performed using backward stepwise regression including the most relevant variables (age, sex, diabetes mellitus, NIHSS at admission, daytime/nighttime, onset-door time (min), [CXCL12] at admission and at 24 h, treatment with IV thrombolysis and mechanical thrombectomy), excluding variables with significance *p* > 0.10. For logistic regression models, discrimination was assessed using the area under the ROC curve (AUC) and calibration with the Hosmer-Lemeshow test. For the continuous outcome, predictive performance is reported using *R*-squared and adjusted *R*-squared.

### Blinding

Although this was a cohort study, patient information was anonymized with a numerical code assigned by chronological order of recruitment. This allowed us to maintain blinding between the different sections of the study (clinical assessment, radiological study and laboratory analysis). The patient's clinical severity, prognosis, or complications could not be determined before the analysis of the samples. Only the previously mentioned data was included in the final statistical analysis.

### Ethical statement

The study was conducted in accordance with the principles of the Declaration of Helsinki. It was approved by the Ethics Committee of “12 de Octubre” University Hospital (reference 18/520). Written informed consent was obtained from all participants prior to inclusion.

Additional Supplemental Methods can be found in Supplementary Material.

## Results

A total of 134 patients were enrolled in the study (mean age, 74.2 ± 14.6 years; female 53.0%). Stroke onset was known in 76.1%, while 19.4% where “wake-up” stroke and 4.5% stroke of unknown onset (<6 h from last seen well); 72.4% of strokes occurred during daylight (8 am–8 pm). The mean NIHSS score was 12.5 (SD ± 7.9). Any intracranial occlusion was observed in 76.9% of patients. Acute reperfusion treatment was performed in 91% of patients: 20.9% with IV thrombolysis, 44.8% with primary mechanical thrombectomy and 25.4% with a combined treatment (thrombolysis + mechanical thrombectomy). The mean infarct volume at 24–72 h was 40 mL (SD ± 66.7), and 24.6% of patients experienced any type of hemorrhagic transformation, but only 6 of these were symptomatic. At three months, 60.5% of patients achieved functional independence (mRS ≤ 2), and 9 patients (6.7%) died during this period. For more details see Tables 1, 2.

**Table 1:** Patient characteristics (demographics, prior conditions, previous treatments, acute-phase treatments), neurological evaluation and radiological work-up at admission, and correlation with CXCL12 levels at admission and 24 h. ASPECTS score analysis was performed in patients with hemispheric stroke who did not present multiple arterial territories infarction, in whom ASPECTS score and CXCL12 levels at admission were available (*N* = 110). Tan scale analysis was conducted in patients with intracranial carotid, M1, or M2 occlusion, in whom both Tan score and CXCL12 concentrations at admission were available (*N* = 69). Admission clinical and radiological parameters were correlated exclusively with CXCL12 levels measured at admission. Linear, ordinal, logistic, or multinomial logistic regression models were applied as appropriate, with results expressed as Odds Ratio (OR), regression coefficient, or Relative Risk Ratio (RRR).

Variable	Total patients *N* = 134	[CXCL12] at admission OR, RRR or *β* (CI 95%) *N* = 121	*P*	[CXCL12] at 24 h OR, RRR or β (CI 95%) *N* = 131	*P*
Demographics					
Age (years), mean (SD), β	74.2 (± 14.6)	3.89 (1.25–6.54)	**<0.01**	3.47 (0.41–6.53)	**0.03**
Women, *n* (%), OR	71 (53.0%)	1.21 (0.84–1.75)	0.31	1.09 (0.72–1.66)	0.68
Medical history					
Arterial Hypertension, *n* (%), OR	87 (64.9%)	1.36 (0.92–2.02)	0.12	1.28 (0.82–2.01)	0.28
Dyslipidemia, *n* (%), OR	74 (55.2%)	1.02 (0.71–1.47)	0.91	0.99 (0.65–1.51)	0.96
Diabetes mellitus, *n* (%), OR	26 (19.4%)	0.48 (0.28–0.81)	**<0.01**	0.52 (0.28–0.96)	**0.03**
Atrial fibrillation, *n* (%), OR	57 (42.5%)	1.03 (0.71–1.49)	0.88	1.00 (0.65–1.52)	0.98
Prior stroke or TIA, *n* (%), OR	14 (10.5%)	1.30 (0.72–2.34)	0.38	1.91 (0.99–3.72)	0.05
Prior intracranial hemorrhage, *n* (%), OR	2 (1.5%)	1.20 (0.29–4.93)	0.80	0.86 (0.15–5.05)	0.87
Prior Myocardial infarct, *n* (%), OR	12 (9.0%)	0.83 (0.43–1.63)	0.60	1.03 (0.51–2.14)	0.92
Peripheric artery disease, *n* (%), OR	3 (2.2%)	1.60 (0.51–5.04)	0.42	1.29 (0.34–4.87)	0.71
Chronic Kidney Disease, *n* (%), OR	12 (9.0%)	1.10 (0.55–2.20)	0.79	0.88 (0.42–1.86)	0.74
Antiplatelet treatment, *n* (%), OR	33 (24.6%)	0.77 (0.50–1.19)	0.24	0.91 (0.56–1.50)	0.72
Anticoagulant treatment, *n* (%), OR	25 (18.7%)	0.99 (0.61–1.60)	0.97	1.23 (0.73–2.07)	0.44
Clinical and imaging at admission					
NIHSS, mean (SD), β	12.5 (±7.9)	−1.26 (−2.69–0.16)	0.08		
ASPECTS (*N* = 110), OR (ordinal logistic regression)		1.62 (1.11–2.37)	**0.01**		
ASPECTS 10, *n* (%)	59 (53.6%)				
ASPECTS 9, *n* (%)	19 (17.3%)				
ASPECTS 8, *n* (%)	12 (10.9%)				
ASPECTS ≤7, *n* (%)	20 (18.2%)				
Collateral circulation (Tan scale) (*N* = 69), OR (ordinal logistic regression) *		1.26 (0.85–1.86)	0.25		
0–1, *n* (%)	21 (30.4%)				
2, *n* (%)	31 (44.9%)				
3, *n* (%)	17 (24.6%)				
Stroke onset					
Diurnal stroke (08–20h), *n* (%), OR	97 (72.4%)	1.22 (0.80–1.86)	0.36	0.96 (0.60–1.54)	0.88
Acute treatment					
Intravenous thrombolysis, *n* (%), OR	62 (46.3%)	0.90 (0.63–1.31)	0.60		
Mechanical thrombectomy, *n* (%), OR	94 (70.1%)	0.76 (0.51–1.15)	0.20		

Bold values indicate statistically significant results (*p* < 0.05).

**Table 2:** Patient neurological and radiological evolution and correlation with CXCL12 levels at admission and 24 h. Linear, ordinal, logistic, or multinomial logistic regression models were applied as appropriate, with results expressed as Odds Ratio (OR), regression coefficient, or Relative Risk Ratio (RRR).

Variable	Total patients *N* = 134	[CXCL12] at admission OR, RRR or β (CI 95%) *N* = 121	*P*	[CXCL12] at 24h OR, RRR or β (CI 95%) *N* = 131	*P*
Radiologic evolution					
Infarct volume (mL), mean (SD), β	40.0 (±66.7)	−11.8 (−24.4–0.85)	0.07	−15.7 (−29.7—−1.63)	**0.03**
Infarct volume (3rd vs. 1st and 2nd tercile), OR		0.74 (0.50–1.10)	0.14	0.52 (0.31–0.85)	**0.01**
Hemorrhagic transformation, *n* (%), OR	33 (24.6%)	0.69 (0.45–1.08)	0.11	0.56 (0.33–0.97)	**0.04**
Symptomatic hemorrhagic transformation, *n* (%), OR	6 (4.5%)	0.67 (0.28–1.59)	0.36	0.68 (0.23–2.03)	0.49
Neurological evolution					
NIHSS at admission, mean (SD), β	12.5 (±7.9)	−1.26 (−2.69–0.16)	0.08		
NIHSS at 24h, mean (SD), β	6.9 (7.3)	−0.88 (−2.26–0.50)	0.21	−1.26 (−2.81–0.28)	0.11
NIHSS at 7d/discharge, mean (SD), β	4.9 (6.4)	−0.63 (−1.93–0.68)	0.34	−0.98 (−2.34–0.38)	0.16
Infection, *n* (%), OR	23 (17.2%)	1.00 (0.63–1.59)	0.99	0.72 (0.39–1.30)	0.27
Functional independence 7d/discharge, *n* (%), OR	57 (57.5%)	1.14 (0.79–1.65)	0.49	1.25 (0.82–1.90)	0.31
Functional independence at 3 months, *n* (%) OR	81 (60.5%)	1.16 (0.80–1.68)	0.44	1.13 (0.74–1.75)	0.57
Death at 3 months, *n* (%), OR	9 (6.7%)	1.35 (0.68–2.70)	0.39	1.08 (0.46–2.56)	0.86
Ethiological subtypes					
Cardioembolic, *n* (%) RRR	65 (48.5%)	Base outcome		Base outcome	
Large-artery atherosclerosis *n* (%) RRR	49 (36.6%)	1.04 (0.70–1.56)	0.82	1.29 (0.82–2.04)	0.28
Arterial dissection, *n* (%) RRR	2 (1.5%)	-		-	
Undetermined cause, *n* (%) RRR	18 (13.4%)	1.23 (0.69–2.20)	0.47	0.69 (0.34–1.42)	0.32

Bold values indicate statistically significant results (*p* < 0.05).

### CXCL12 and stroke outcome

**Figure 1:**
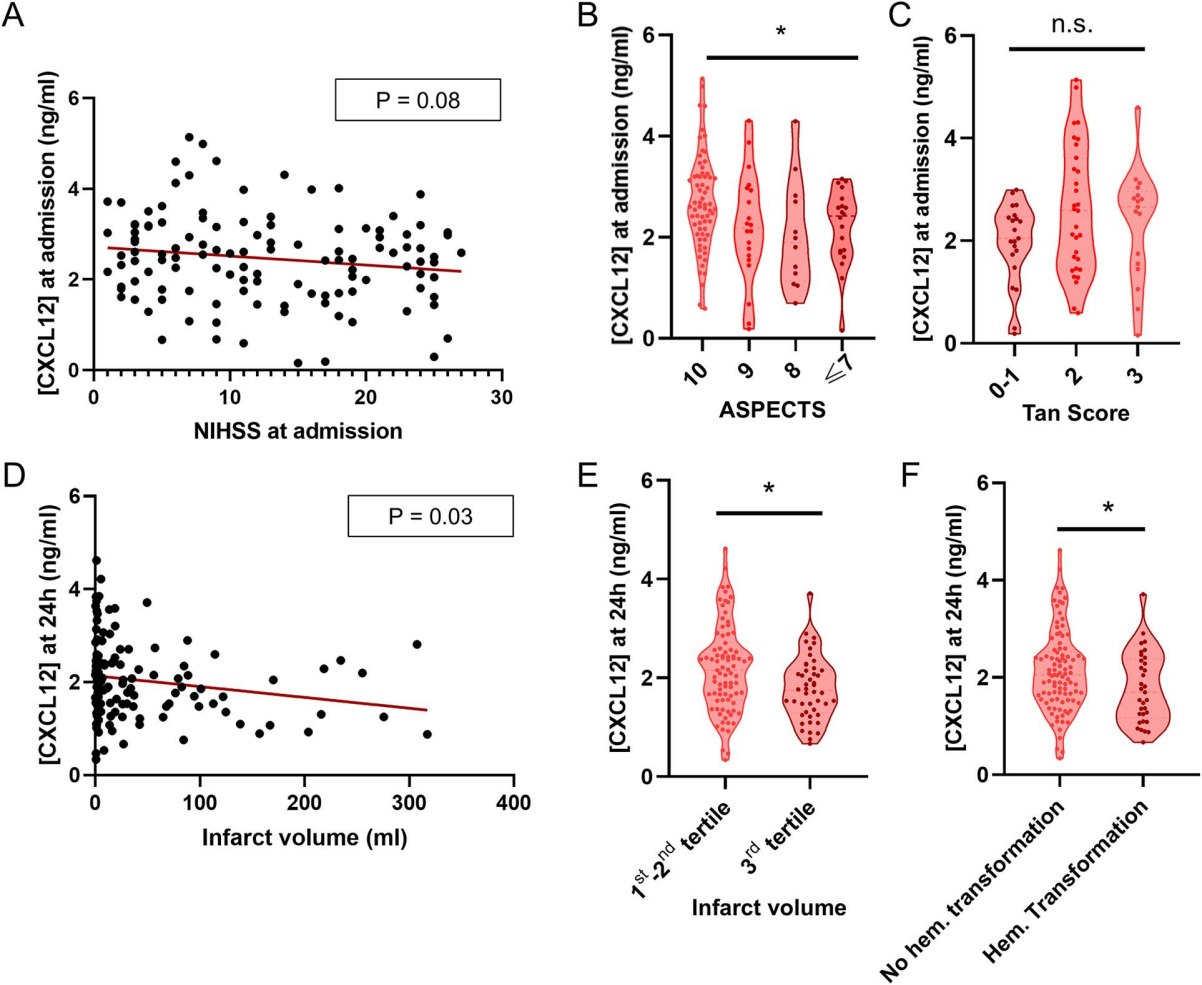
CXCL12 and stroke outcome. **(A)** Lack of correlation between NIHSS on admission and plasma CXCL12 levels at admission. **(B)** Violin plot showing significant differences between ASPECTS score and CXCL12 at admission (*P* = 0.01, ordinal logistic regression analysis). This analysis was performed in patients with hemispheric stroke who did not present multiple arterial territories infarction for whom both ASPECTs score and CXCL12 concentrations at admission were available (*N* = 110). **(C)** Violin plot showing plasma CXCL12 levels on admission in Tan score (*P* > 0.05). This analysis was focused on patients with intracranial carotid, M1, or M2 occlusion for whom both Tan score and CXCL12 concentrations at admission were available (*N* = 69). **(D)** Scatterplot showing a negative correlation between infarct volume at 24–72 h and CXCL12 levels at 24 h (±12 h); infarct volume was measured in mL (*P* = 0.03, linear regression analysis). **(E)** Violin plot showing significant differences in CXCL12 levels at 24 h (±12h) between the upper tertile of infarct volume with the rest of the patients (*P* = 0.01, logistic regression analysis). **(F)** Violin plot showing significant differences between CXCL12 levels at 24 h (±12 h) and hemorrhagic transformation (any type) (*p*=0.04, logistic regression analysis).

No significant differences were observed between CXCL12 concentrations at admission and clinical severity measured by the NIHSS scale, although higher CXCL12 levels were associated with a trend toward reduced clinical severity (Figure 1A). However, higher CXCL12 levels correlated with lower radiological severity, as measured by the ASPECTS scale (*P* = 0.01, Figure 1B), while no significant differences were found in collateral circulation assessed by the Tan scale (*P* = 0.28, Figure 1C).

**Figure 2:**
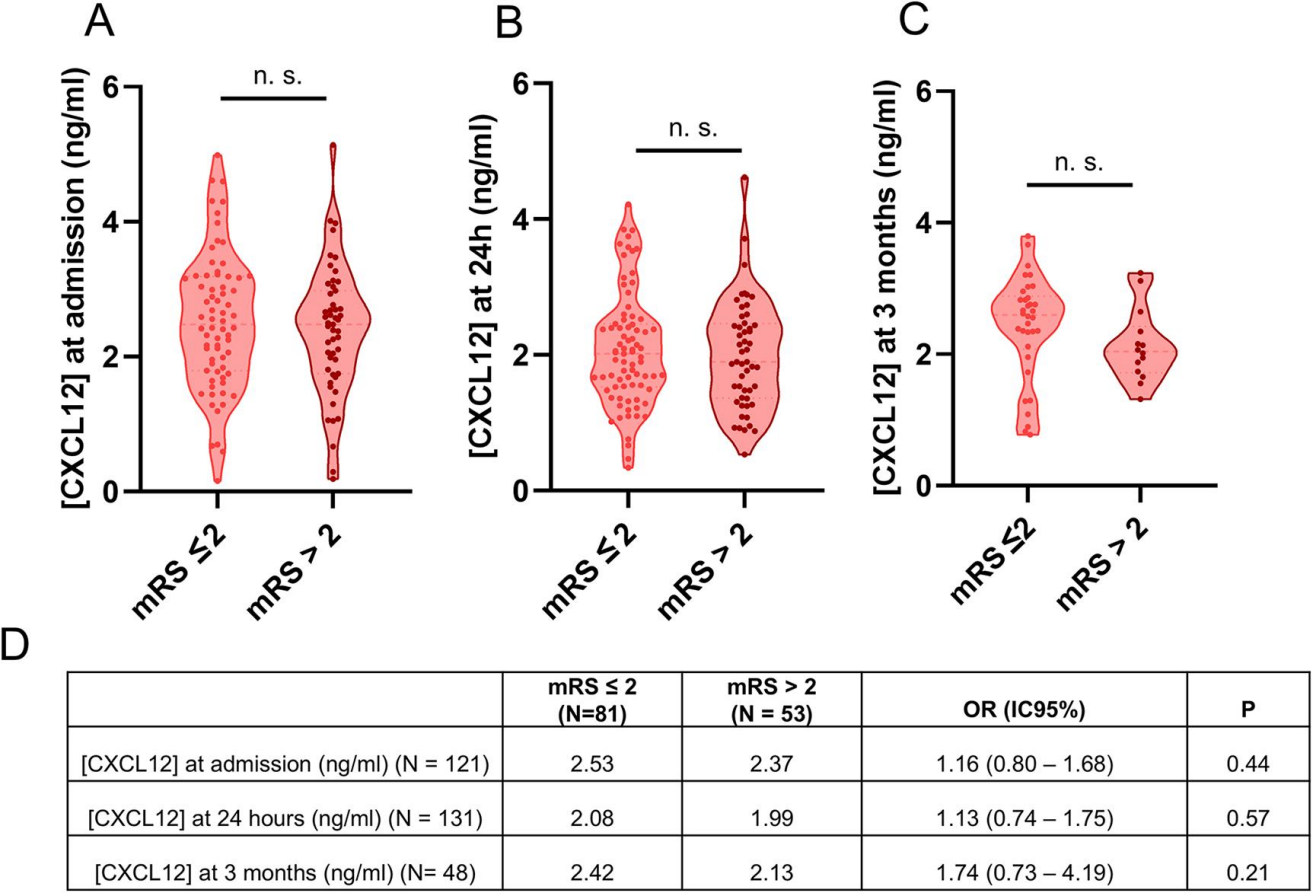
CXCL12 at admission, 24 h and 3 months and functional prognosis at three months. Plasma CXCL12 concentration at admission **(A)**, at 24 h **(B)**, and at 3 months **(C)** and the likelihood of achieving functional independence at three months. **(D)** The sample is divided into two groups: patients with functional independence (mRS ≤ 2) and those who are dependent or deceased (mRS > 2). Data is represented using a violin plot. Analysis was performed using logistic regression (n.s, not significant).

Notably, a significant negative correlation was observed between plasma CXCL12 levels 24 h after stroke and infarct volume, both in direct ischemic area analysis (*P* = 0.03, Figure 1D) and when categorized into tertiles (*P* = 0.01, Figure 1E). Additionally, higher CXCL12 levels at 24 h were significantly associated with a lower likelihood of hemorrhagic transformation (*P* = 0.04, Figure 1F). However, no association was identified between the levels CXCL12, measured at admission, 24 h and three months, and the functional prognosis at three months, as determined by the mRS (Figures 2A–C). These results did not differ significantly when patients with posterior circulation were excluded from the analysis. For more details see Table 2.

### CXCL12, age and diabetes

**Figure 3:**
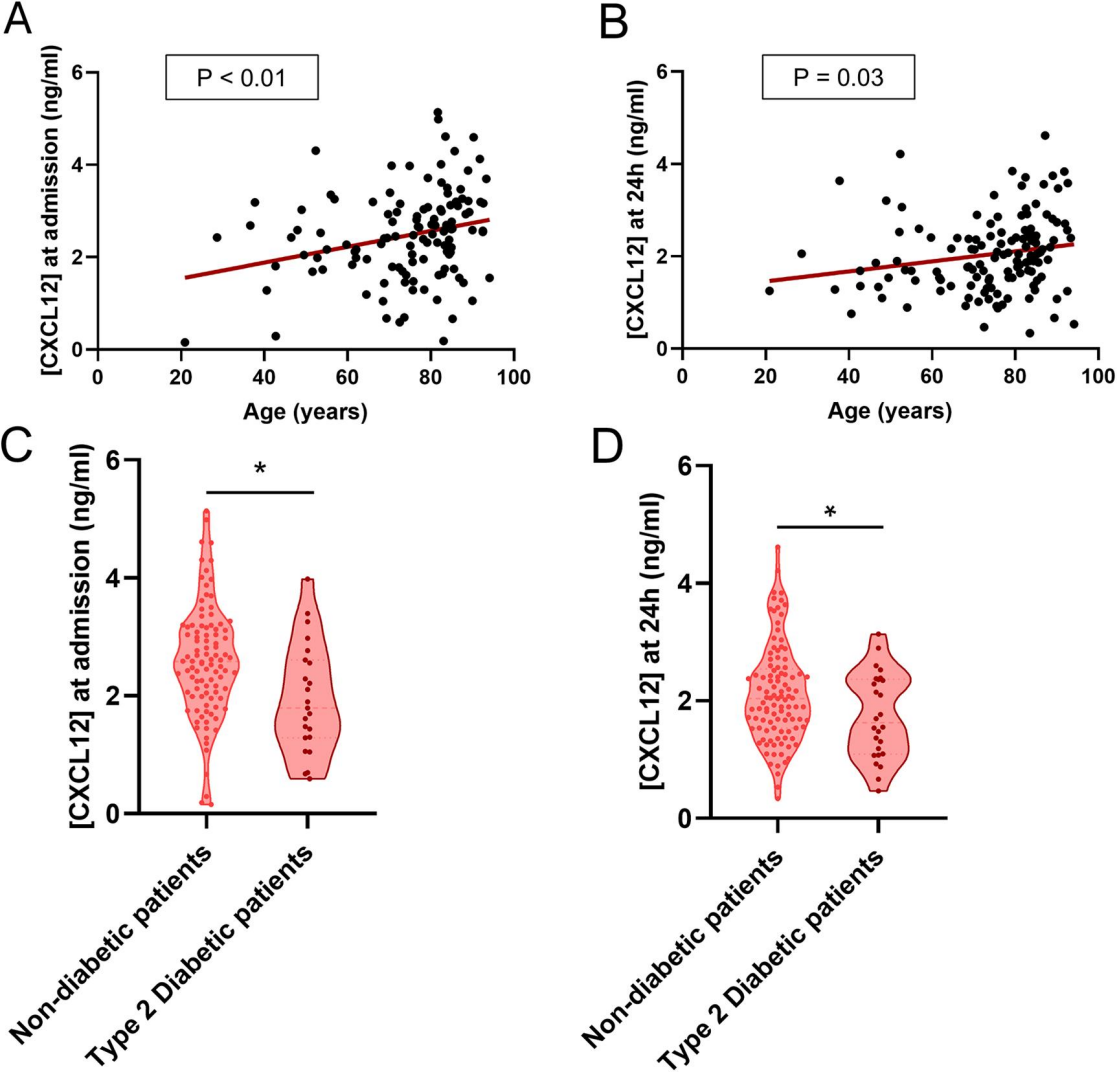
CXCL12, age, and diabetes. **(A,B)** Positive correlation between plasma CXCL12 levels at admission **(A)** and at 24 h **(B)** and age. **(C,D)** Violin plot showing significant differences between non-diabetic and diabetic patients in plasma CXCL12 levels at admission **(C)** and at 24 h **(D).**

Subsequently, we aimed to investigate the potential influence of key demographic variables and pre-existing comorbidities on CXCL12 levels, an area that remains unexplored in prior studies. In our cohort, CXCL12 levels showed a positive correlation with age at both admission (*p* < 0.01) and 24 h (*p* = 0.03) (Figures 3A,B). Non-diabetic patients had significantly higher CXCL12 levels (*P* < 0.01 at admission; *P*=0.03 at 24 h, Figures 3C,D). No other notable associations with other pathologies were identified; full details are provided in Table 1.

### Predictive model and CXCL12

To evaluate the influence of CXCL12 on the prognosis of stroke patients, a predictive study was conducted using a backwards stepwise model, incorporating the main factors that may affect clinical and radiological outcomes, including stroke severity at admission, infarct volume, hemorrhagic transformation, and functional independence at three months. As previously reported, higher NIHSS at admission and diabetes mellitus were strongly associated with larger infarct volumes, worse clinical outcomes, and increased risk of bleeding complications. Notably, elevated CXCL12 levels at 24 h appeared to have a protective effect by reducing infarct volume. These findings underscore the potential of CXCL12 as a prognostic biomarker (Table 3).

**Table 3:** Predictive analysis. A Predictive study was performed using *backward stepwise* regression including the most relevant variables (age, sex, diabetes mellitus, NIHSS at admission, daytime/nighttime, onset-door time (min), [CXCL12] at admission and at 24 h, treatment with IV thrombolysis and mechanical thrombectomy), excluding variables with significance *p* > 0.10. For logistic regression models, discrimination was assessed using the area under the ROC curve (AUC) and calibration with the Hosmer-Lemeshow test. For the continuous outcome, predictive performance is reported using *R*-squared and adjusted *R*-squared. The predictive analysis to determine NIHSS at admission did not include variables for acute phase treatment (IV fibrinolysis or mechanical thrombectomy) or plasma CXCL12 concentration at 24 h.

Variable	β coefficient or Odds Ratio (OR) (IC95%)	*P* value
NIHSS at admission, β		
Male sex	−2.75 (−5.53–0.03)	0.05
[CXCL12] at admission (ng/mL)	−1.18 (−2.60–0.23)	0.10
R^2^ 0.06; adjusted R^2^ 0.04		
Infarct volume (mL), β		
NIHSS at admission	3.95 mL (2.52–5.38)	<0.01
Diabetes mellitus	33.57mL (4.93–62.21)	0.02
R^2^ 0.25; adjusted R^2^ 0.24		
Infarct volume, (3rd tertile vs. 1st and 2nd tertile), OR		
NIHSS at admission	1.15 (1.08–1.23)	<0.01
Onset to admission time (min)	1.008 (1.002–1.014)	0.02
[CXCL12] at 24 h (ng/mL)	0.52 (0.28–0.97)	0.04
AUC (95% CI) 0.80 (0.72–0.88); Hosmer-Lemeshow test (*p*) = 0.68		
Hemorrhagic transformation (any type), OR		
Mechanical thrombectomy	7.62 (1.68–34.51)	<0.01
Diabetes mellitus	2.80 (1.01–7.77)	0.048
AUC (95% CI) 0.70 (0.62–0.79); Hosmer-Lemeshow test (*p*) = 0.29		
Functional independence at 3 months (mRS ≤ 2), OR		
Age (years)	0.95 (0.92–0.99)	<0.01
NIHSS at admission	0.86 (0.81–0.92)	<0.01
AUC (95% CI) 0.81 (0.73–0.89); Hosmer-Lemeshow test (*p*) = 0.25		

### CXCL12 and neutrophil-related parameters

**Figure 4:**
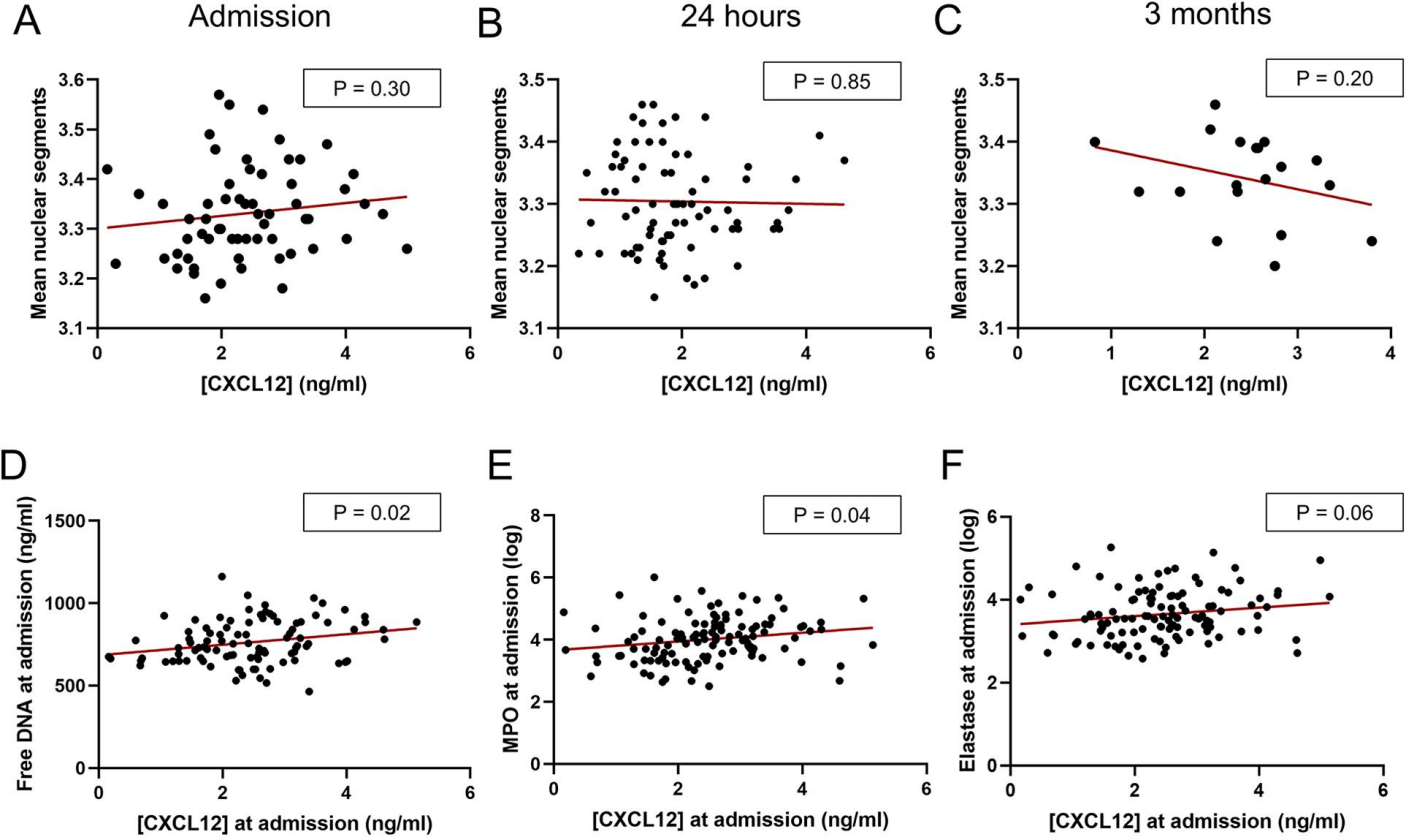
CXCL12 and neutrophil-related parameters. **(A–C)** Scatterplot showing no correlation between plasmatic CXCL12 levels and neutrophil nuclear segmentation at admission (<6 h after stroke onset) **(A)**, *n* = 61, 24 h (±12 h) **(B)**, *n* = 77, and 3 months **(C)**, *n* = 19. **(D–F)** Scatterplot between plasmatic CXCL12 levels and plasmatic NET markers at admission: **(D)**: Free DNA; **(E)**: myeloperoxidase (MPO); and **(F)**: neutrophil elastase. All data were analyzed by linear regression.

Finally, an exploratory study was carried out to investigate whether the effect of CXCL12 on the prognosis of ischemic stroke patients could be mediated by neutrophil phenotype or activity. No significant differences in neutrophil phenotype were observed based on the time of ischemic stroke onset or plasma CXCL12 concentration when assessing neutrophil aging through nuclear segmentation (Figures 4A–C). However, at admission, plasma CXCL12 levels showed a positive correlation with plasma NETs markers, including free DNA, myeloperoxidase (MPO) and neutrophil elastase (NE). This correlation was statistically significant for all markers except NE (Figures 4D–F).

## Discussion

This study shows a significant correlation between CXCL12 levels and acute stroke outcomes, specifically regarding radiological severity, infarct volume, and risk of hemorrhagic transformation. Higher CXCL12 concentrations were associated with reduced infarct size and lower hemorrhagic transformation risk, suggesting CXCL12 may play a cerebroprotective role in the acute phase of ischemic stroke.

The role of CXCL12 in stroke remains complex, with contradictory findings suggesting it may be context-dependent. As regards the observed actions in the acute phase, preclinical studies have reported cerebroprotective effects of CXCL12: early research in animal models of ischemic stroke observed increased CXCL12*β* expression in endothelial cells of penumbral blood vessels associated with a concomitant infiltration of CXCR4-expressing peripheral blood cells (17). In neuronal cultures, CXCL12 administration demonstrated cerebroprotection against neurotoxicants like H₂O₂, additionally, in a murine ischemic stroke model, CXCL12*α* administration reduced infarct volume (18). CXCL12/CXCR4 axis has also been associated with improved prognosis mediated by mesenchymal stem cells (19), monocytes (20), NK cells (21) and endothelial progenitor cells (EPCs) (22). In contrast, inhibition of CXCR4 has also shown benefits, including microglia-mediated cerebroprotection (23) and reduced lymphocyte infiltration (24).

Clinical studies on CXCL12 in acute stroke have also produced conflicting results, varying by population. Research on Caucasian cohorts has linked higher plasma CXCL12 levels to smaller infarct volumes (25, 26) in agreement with our data. Conversely, studies in Asian populations have associated elevated CXCL12 levels with larger infarct volumes and worse neurological outcomes (27, 28). Additionally, one study found that higher CXCL12 levels correlated with poorer neurological outcomes in univariate analysis. However, after adjusting for NIHSS and infarct volume in multivariate analysis, elevated CXCL12 levels were instead linked to a higher likelihood of favorable long-term outcomes (29). These discrepancies may stem from population-specific genetic or epigenetic factors affecting CXCL12 regulation and signaling, highlighting the need for further population-based research.

The observed relationship between plasma CXCL12 levels and the likelihood of hemorrhagic transformation warrants further investigation. One of the factors that may influence this is the infarct volume itself, it is expected that patients with a larger infarct volume and those with lower CXCL12 concentrations will have a higher rate of hemorrhagic transformation. However, it can also be considered that CXCL12 may have a direct effect on the endothelium, as it is one of the chemokines involved in angiogenesis and the migration of endothelial progenitor cells (25). In addition, CXCL12 may modulate local inflammatory activity, potentially contributing to or preventing blood–brain barrier disruption.

While we observed correlations between CXCL12 levels and acute stroke severity markers, causal relationships remain speculative. In this context, it is important to consider that elevated CXCL12 levels may not necessarily act as a direct causal factor leading to improved outcomes but could instead reflect a reactive or compensatory biological response to ischemic injury. CXCL12 is rapidly upregulated in the ischemic brain and vasculature and has been linked to endogenous repair mechanisms, including angiogenesis, recruitment of endothelial progenitor cells, and neurovascular remodeling (17, 18, 22, 25). From this perspective, higher circulating CXCL12 levels may indicate preserved reparative capacity or smaller infarct burden rather than directly mediating cerebroprotection. This dual role of CXCL12 as both a potential effector and a biomarker of tissue response may partly explain the heterogeneous findings reported across clinical studies (25–29).

This study identified a positive correlation between age and plasma CXCL12 levels, both at admission and at 24 h, with higher CXCL12 concentrations observed in older patients. Similar age-related increases in CXCL12 have been reported in other pathological contexts: higher circulating CXCL12 levels have been described in older patients with osteoporosis, and comparable trends have been observed at the tissue level in the thymus and prostate (30–32). Although the underlying pathophysiological mechanisms linking age to CXCL12 production remain unclear, one possible hypothesis is that higher CXCL12 levels may be associated with reduced vascular damage, thereby delaying the occurrence of vascular events to later ages or manifesting in a less severe form. In the context of stroke, this age-related increase in CXCL12 may partially contribute to the lack of differences observed in 3-month functional outcomes, given that age is a major determinant of poor prognosis. While the relationship between age and CXCL12 has not been previously explored in stroke patients, the CXCR4/CXCL12 axis has been implicated in aging-related processes such as immunosenescence, which may influence CXCL12 expression or function (33).

In addition, CXCL12 plasma concentrations were significantly lower in patients with diabetes mellitus. Previous studies have reported heterogeneous effects of CXCL12 in diabetes depending on the stage of the disease. Elevated CXCL12 levels may promote pancreatic *β*-cell regeneration and proliferation, suggesting a protective role in the prevention of type 1 diabetes mellitus (34). Furthermore, a recent murine study showed that mice with constitutively young neutrophils (CXCR2^loxP/MRP8Cre+) exhibit increased insulin sensitivity (35), supporting a potential protective role of some neutrophil subtypes in the pathogenesis of diabetes. However, once diabetes is established, CXCL12-driven angiogenesis may exert deleterious effects in specific situations, such as diabetic retinopathy or nephropathy (34). Nevertheless, it remains unclear whether, after diabetes onset, CXCL12 may induce alterations in cerebral circulation that could influence stroke prognosis. Further research is therefore warranted to clarify how age and diabetes modulate the effects of CXCL12 across different stroke patients' subgroups.

Now, our study adds to the literature by examining the relationship between CXCL12 and neutrophil phenotype, as well as its association with NETs markers. While no significant association was observed between CXCL12 levels and neutrophil phenotype assessed by nuclear segmentation, we did find a positive correlation between CXCL12 and plasmatic NETs markers that could be associated with differences in neutrophil activity. Conversely, NETosis has been implicated in exacerbating damage in the ischemic brain and other tissues (36, 37). In this context, several pieces of evidence support conflicting views. Previous studies have shown that young neutrophils, favored by the CXCR4-CXCL12 axis, display an increased capacity for NET formation. However, despite this enhanced NETotic potential, this neutrophil phenotype has been associated with reduced myocardial injury in murine models of myocardial infarction, showing the complex and context-dependent balance of immune responses in vascular diseases (11, 13). Moreover, studies using murine models have observed decreased transcription of NETosis-associated genes (MPO, NE, PADI4) following administration of AMD3100, a CXCR4 antagonist (38). In contrast, during ischemic stroke, the presence of NETs has been associated with poor prognosis (39).

The positive association observed in our study between CXCL12 and circulating NETs markers may therefore appear counterintuitive. Currently, mechanistic data specifically addressing the balance between CXCL12 signaling and NETosis in ischemic stroke have not been previously described. Future studies including preclinical models should explore this relationship longitudinally to determine whether this association persists beyond the hyperacute phase. It should also be investigated whether CXCL12 may exert an independent protective effect in this context, potentially modulating the immune response to cerebral ischemia. Finally, as NETosis was assessed indirectly through plasma biomarkers, further studies are needed to evaluate if CXCL12 could modify local intravascular NET formation or within the affected brain parenchyma. All these findings highlight the need for further research into how CXCL12 can influence neutrophil responses in ischemic stroke (including NETosis), potentially influencing the balance between brain cerebroprotection and inflammation.

This is the first study to explore CXCL12’s role in stroke outcomes in a cohort of patients who may be treated with mechanical thrombectomy, allowing us to investigate its association with infarct volume and hemorrhagic transformation under current treatment standards. Another strength of this study is the selection of patients with a shorter stroke evolution time compared to other studies—less than 6 h instead of 24 h. This narrower timeframe may reduce variability in the inflammatory response, providing a more consistent baseline for evaluating acute-phase biomarkers.

Despite these promising findings, our study has limitations. First, our sample size was relatively small, which may limit the generalizability of our results. Larger, multicenter studies including multi-ethnic cohorts are needed to validate our findings and explore potential subgroup effects. This study was intended to generate hypotheses and provide preliminary evidence for further studies. Second, our study design did not allow for continuous monitoring of CXCL12 levels throughout the subacute and chronic phases, limiting our ability to assess its potential role beyond the acute phase of stroke. Additionally, we relied on indirect measurements of neutrophil aging based on nuclear segmentation; future studies might benefit from incorporating flow cytometry-based assessment of surface markers (e.g., CXCR2, CD62L, and CXCR4) to more accurately define neutrophil subsets and achieve a more precise phenotypic characterization. Finally, while we observed correlations between CXCL12 levels and acute stroke severity markers, causal relationships remain speculative. It is possible that CXCL12 could modify the stroke-induced inflammatory response through neutrophils or the neurovascular unit rather than a direct contributor to reduced infarct severity. Experimental studies focused on CXCL12 modulation in animal models or with pharmacological agents targeting the CXCR4/CXCL12 axis could provide further insights.

In summary, our study suggests that CXCL12 levels are associated with reduced infarct volume and a lower risk of hemorrhagic transformation in acute ischemic stroke. These findings support the potential role of CXCL12 as a biomarker associated with stroke severity. Our study contributes to understanding the potential associations between CXCL12 and ischemic stroke, particularly regarding acute-phase severity and opens new areas of research in NETs-associated inflammation. Further research is warranted to clarify the mechanisms by which CXCL12 can modify the neutrophil's phenotype and explore potential therapeutic interventions**.**

## Data Availability

The raw data supporting the conclusions of this article will be made available by the authors, without undue reservation.
