# Supplementary material for: CXCL12 levels correlate with reduced stroke severity and lower risk of hemorrhagic transformation in stroke patients
Source: Front Cardiovasc Med. 2026 May 12;13:1753482. doi: 10.3389/fcvm.2026.1753482 (PMC13201131; doi:10.3389/fcvm.2026.1753482)
Supplement: Supplementary file 1 [file Supplementaryfile1.pdf]

Supplementary Materials and Methods

Patient inclusion and exclusion criteria

1. Inclusion criteria

- Age ≥18 years.
- Good functional status before stroke, defined by a score of ≤2 on the modified Rankin scale, which ranges from 0 [no neurologic deficit] to 6 [death].
- Ischemic stroke of ≤ 6 hours duration or "wake-up" stroke. In the case of a wake-up stroke, no more than 6 hours should have elapsed from the midpoint between sleep onset and time of waking until hospital admission.
- Patient, family member, or legally responsible person, has given informed consent.

2. Exclusion criteria

- Lacunar stroke.
- Infectious disease in the previous 15 days. During the COVID-19 outbreak in 2020, patient recruitment was temporarily interrupted for two months (February 24th to April 28th). After recruitment resumed, COVID-19 screening was initially performed using chest CT imaging to rule out pneumonia, followed by a nasopharyngeal PCR test to exclude asymptomatic infection.
- Severe systemic disease, including terminal renal disease or severe liver disease.
- Systemic inflammatory disease, central nervous system vasculitis or patient receiving immunosuppressive therapy.
- Active cancer disease or chemotherapy treatment.

- Transient ischemic attack (TIA) or ischemic stroke without a visible ischemic lesion on CT scan or MRI.

Sample size and data analysis

This study was designed as an exploratory analysis, and therefore no formal a priori sample size or power calculation was performed. A target of more than 100 patients was instead predefined at the design stage based on feasibility and expected patient flow.

We focused on presenting effect sizes and confidence intervals rather than solely p-values to facilitate clinical interpretation, formal statistical corrections for multiple comparisons (e.g., Bonferroni) were not applied.

Radiologic work-up.

1. CT scan and AngioCT at admission.

Upon admission, all patients underwent a CT scan, in which the Alberta Stroke Program Early Computed Tomography Score (ASPECTS) was measured. ASPECTS values range from 0 to 10, with higher values indicating less infarct burden (1). ASPECTS score analysis was performed in patients with hemispheric stroke who did not present multiple arterial territories infarction and ASPECTS score could be calculated (N = 120).

To assess collateral circulation in AngioCT at admission, we used Tan's scale, which rates the degree of collateral circulation on a scale of 0 to 3 (higher values indicate a better collateral status). Due to the small number of patients with a grade of 0, patients with grades 0 and 1 have been combined into the same category (2). This analysis was focused on patients with intracranial carotid, M1,

or M2 occlusion (N = 93) for whom both Tan score and CXCL12 concentrations at admission were available (N = 69). This approach was used to standardize collateral assessment and minimize potential bias in patients in whom collateral status cannot be reliably evaluated (e.g., non/distal/medium-caliber artery occlusions).

2. Mechanical Thrombectomy

Patients' acute treatment was performed as established in ESO guidelines. If mechanical thrombectomy was indicated, arterial recanalization was measured with modified treatment in cerebral infarction **(**mTICI**)** score (3), including TICI2c (4)**.**

- Grade 0: no perfusion.
- Grade 1: antegrade reperfusion past the initial occlusion, but limited distal branch filling with little or slow distal reperfusion.
- Grade 2:
  - Grade 2a: antegrade reperfusion of less than half of the occluded target artery previously ischemic territory (e.g. in one major division of the middle cerebral artery (MCA) and its territory).
  - Grade 2b: antegrade reperfusion of more than half of the previously occluded target artery ischemic territory.
  - Grade 2c: near complete perfusion except for slow flow or distal emboli in a few distal cortical vessels.
- Grade 3: complete antegrade reperfusion of the previously occluded target artery ischemic territory, with absence of visualized occlusion in all distal branches.

3. Determination of infarct size

The volume of cerebral infarction was determined with a CT scan or MRI at 24-72 hours. Infarcted area was first quantified in each image (cm^2), being infarct volume obtained (cm^3) as the sum of the orthogonal projections of each damaged area over the slice thickness. If infarct volume was measured using cranial MRI, the diffusion sequence (DWI) was utilized. The decision to perform a CT scan or MRI was based solely on clinical criteria, depending on what is expected in each patient to complete the etiological study.

4. Hemorrhagic transformation

In the neuroradiological study conducted to measure cerebral infarction, it was determined whether a hemorrhagic transformation had occurred.

The classification was based on the presence or absence of any type of hemorrhagic transformation, regardless of its severity or location.

If the patient has experienced symptomatic hemorrhagic transformation according to the criteria of the ECASS III study (5), meaning that it has been the main identified cause for the patient's significant clinical deterioration (worsening ≥ 4 points on the NIHSS scale or resulting in the patient's death).

Parenchymal hemorrhagic transformations was classified as follows: HI-1 is defined as small petechiae along the margins of the infarct, HI-2 represents more confluent petechiae within the infarcted area, but without space-occupying effect, PH-1 is defined as blood clot not exceeding 30 percent of the infarcted area with some mild space-occupying effect, and PH-2 represents dense blood clot exceeding 30 percent of the infarct volume with significant space-occupying

effect. Additionally, it has been analyzed whether the patient has presented a hemorrhagic transformation in another neurovascular territory (also known as remote hemorrhage).

For patients with subarachnoid hemorrhage, it has been quantified as either focal (adjacent to the area of cerebral infarction) or diffuse.

REFERENCES

1. Barber PA, Demchuk AM, Zhang J, Buchan AM. Validity and Reliability of a Quantitative Computed Tomography Score in Predicting Outcome of Hyperacute Stroke before Thrombolytic Therapy. Aspects Study Group. Alberta Stroke Programme Early Ct Score. *Lancet* (2000) 355(9216):1670-4. doi: 10.1016/s0140-6736(00)02237-6.

2. Tan IY, Demchuk AM, Hopyan J, Zhang L, Gladstone D, Wong K, et al. Ct Angiography Clot Burden Score and Collateral Score: Correlation with Clinical and Radiologic Outcomes in Acute Middle Cerebral Artery Infarct. *AJNR Am J Neuroradiol* (2009) 30(3):525-31. Epub 20090115. doi: 10.3174/ajnr.A1408.

3. Zaidat OO, Yoo AJ, Khatri P, Tomsick TA, von Kummer R, Saver JL, et al. Recommendations on Angiographic Revascularization Grading Standards for Acute Ischemic Stroke: A Consensus Statement. *Stroke* (2013) 44(9):2650-63. Epub 20130806. doi: 10.1161/STROKEAHA.113.001972.

4. LeCouffe NE, Kappelhof M, Treurniet KM, Lingsma HF, Zhang G, van den Wijngaard IR, et al. 2b, 2c, or 3: What Should Be the Angiographic Target for Endovascular Treatment in Ischemic Stroke? *Stroke* (2020) 51(6):1790-6. Epub 20200513. doi: 10.1161/STROKEAHA.119.028891.

5. Hacke W, Kaste M, Bluhmki E, Brozman M, Dávalos A, Guidetti D, et al. Thrombolysis with Alteplase 3 to 4.5 Hours after Acute Ischemic Stroke. *N Engl J Med* (2008) 359(13):1317-29. doi: 10.1056/NEJMoa0804656.
